# Detection of antibiotic resistance in probiotics of dietary supplements

**DOI:** 10.1186/s12937-015-0084-2

**Published:** 2015-09-14

**Authors:** Aloysius Wong, Davey Yueh Saint Ngu, Lydia Annabel Dan, Amanda Ooi, Renee Lay Hong Lim

**Affiliations:** 1 Division of Biological and Environmental Sciences and Engineering, 4700 King Abdullah University of Science and Technology, Thuwal, 23955-6900 Saudi Arabia; 2 UCSI University, No. 1, Jalan Menara Gading, UCSI Heights, Cheras, 56000 Kuala Lumpur, Malaysia

## Abstract

**Background:**

Probiotics are live microorganisms that confer nutrition- and health-promoting benefits if consumed in adequate amounts. Concomitant with the demand for natural approaches to maintaining health is an increase in inclusion of probiotics in food and health products. Since probiotic bacteria act as reservoir for antibiotic resistant determinants, the transfer of these genes to pathogens sharing the same intestinal habitat is thus conceivable considering the fact that dietary supplements contain high amounts of often heterogeneous populations of probiotics. Such events can confer pathogens protection against commonly-used drugs. Despite numerous reports of antibiotic resistant probiotics in food and biological sources, the antibiogram of probiotics from dietary supplements remained elusive.

**Findings:**

Here, we screened five commercially available dietary supplements for resistance towards antibiotics of different classes. Probiotics of all batches of products were resistant towards vancomycin while batch-dependent resistance towards streptomycin, aztreonam, gentamycin and/or ciprofloxacin antibiotics was detected for probiotics of brands Bi and Bn, Bg, and L. Isolates of brand Cn was also resistant towards gentamycin, streptomycin and ciprofloxacin antibiotics. Additionally, we also report a discrepancy between the enumerated viable bacteria amounts and the claims of the manufacturers.

**Conclusions:**

This short report has highlighted the present of antibiotic resistance in probiotic bacteria from dietary supplements and therefore serves as a platform for further screenings and for in-depth characterization of the resistant determinants and the molecular machinery that confers the resistance.

**Electronic supplementary material:**

The online version of this article (doi:10.1186/s12937-015-0084-2) contains supplementary material, which is available to authorized users.

## Findings

### Introduction

Probiotics are live microorganisms usually bacteria or yeasts, that confer health-promoting benefits to the host if consumed in adequate amounts. These benefits range from improvement of intestinal health [1] and immune response [2], to prevention of acute and antibiotic-associated diarrhea [1], and cancer [3]. The health-promoting attributes, mechanisms and strain-specific benefits have been extensively reviewed elsewhere [4]. Probiotic foods have gained widespread acceptance and popularity [5, 6] as reflected by an estimated growth of more than 10 % in economic value from 2009 to 2014 [7]. Due to increasing consumer demand for natural non-drug approaches to improve health, probiotic bacteria have been included in overwhelming number of food products especially in dairy foods such as the probiotic-containing yoghurt, cheese, milk and ice-cream [8].

Widespread use of probiotic bacteria in conjunction and in close association with antibiotic use or rather misuse, can over time establish a reservoir of antibiotic resistant genes in probiotic bacteria [9]. While intrinsic antibiotic resistance can be a desirable trait as probiotics help restore host gut microflora during a course of antibiotics, however the transfer of resistant genes to pathogenic bacteria offers serious clinical threats [10]. There is already a wide collection of literature reporting resistance of lactic acid bacteria towards antibiotics of the beta-lactams, macrolide, aminoglycoside, chloramphenicol and tetracycline classes [6, 11] and in some, their corresponding resistant genes and mechanisms have been characterized [9, 12]. Importantly, resistant gene transfer that occurs at high frequency and persist across generations have also been demonstrated *in vitro* and/or *in vivo* among *Lactobacilli* and from *Lactobacilli* to pathogens [13] and vice versa [14]. These reports have established probiotic bacteria as reservoir of antibiotic resistant genes that can be transferred to pathogenic strains.

While the detection of antibiotic resistance in probiotic strains from food and biological sources have intensified, such reports from dietary supplements have remained somewhat elusive. This is surprising considering the fact that probiotic dietary supplements contain high amount and often a heterogeneous population of probiotic bacteria both of which, are conditions that encourage the trafficking of resistant genes. Therefore, we hypothesize that antibiotic resistant probiotics may be present in dietary supplements.

### Methods

Probiotic dietary supplements designated here as Bi, Bn, Bg, Cn and L, were purchased from local pharmacy or retail outlets (Table 1) and the ampicillin, aztreonam, erythromycin, gentamycin, streptomycin, clindamycin, vancomycin, ciprofloxacin, cephalexin and tetracycline antibiotic discs were purchased from HiMedia, India (Table 2). In order to recover and enumerate probiotic bacteria [15], one capsule of dietary supplement was dissolved in sterile double distilled water [16] and immediately platted on the de Man, Rogosa and Sharpe (MRS) media (Difco, USA) which is selective for *Lactobacilli*. We note that *Lactobacilli* constitute the majority of the probiotic bacteria population in the dietary supplements (Table 1) and have therefore excluded contributions of other probiotic strains from the bacteria count and from the subsequent antibiotic resistance screening (Additional file 1). One capsule contains equal amounts of each probiotic strain. In products where such information is not stated, equal contributions from each probiotic content is assumed. The dissolved samples (10^6^ CFU) were cultured overnight in MRS broth for enrichment of probiotic bacteria after which the overnight culture was adjusted to uniform concentrations of 7×10^6^ CFU/mL of bacteria by spectroscopy (OD_690nm_) prior to antibiotic susceptibility tests using commercial antibiotic discs and according to the manufacturer’s instructions (Additional file 1).

**Table 1:** The information of probiotic bacteria in dietary supplements

Product	Country manufactured	Probiotic content	Probiotic amount (×10^6^ CFU/capsule)
Bi	Malaysia	*B. longum*, *B. bifidum*,	5,000*
		*B. infantis*, *L. bulgaricus*,	
		*L. rhamnosus*, *L. casei,*	
		*L. acidophilus*	
Bn	Austria	*B. longum*, *B. bifidum,*	10
		*L. gasseri*	
Bg	Malaysia	*L. acidophilus*,	2,000*
		*L. rhamnosus,*	
		*B. longum*	
Cn	U.S.A	*L. acidophilus*,	8
		*L. salivarius*, *B. bifidum,*	
		*S. thermophiles,*	
L	Malaysia	*L. acidophilus*,	10,000*
		*L. bulgaricus*, *L. casei*,	
		*B. longum,*	
		*S. thermophilus*	

*One capsule contains equal amounts of each probiotic strain. In products where such information is not stated, equal contributions from each probiotic content is assumed

**Table 2:** Classification and mode of action of antibiotics

Antibiotic	Class	Spectrum
Ampicillin (10 mcg)	Semi-synthetic beta lactams	Gram positive and Gram negative
Aztreonam (30 mcg)	Mono-bactams	Gram positive and Gram negative
Erythromycin (15 mcg)	Macrolides	Gram positive and Gram negative
Gentamicin (5 mcg)	Aminoglycosides	Gram positive and Gram negative, especially *Pseudomonas*
Streptomycin (10 mcg)	Aminoglycosides	Gram positive and Gram negative
Clindamycin (2 mcg)	Lincomycins	Gram positive and Gram negative, especially anaerobic *Bacteroides*
Vancomycin (30 mcg)	Glycopeptides	Gram positive and Gram negative, especially *S. aureus*
Cephalexin (30 mcg)	Cephalosporin	Gram positive
Tetracycline (30 mcg)	Tetracycline	Gram positive and Gram negative
Ciprofloxacin (10 mcg)	Fluoroquinolones	Gram positive and Gram negative, especially *Bacillus anthracis*

### Results and discussion

**Fig. 1:**
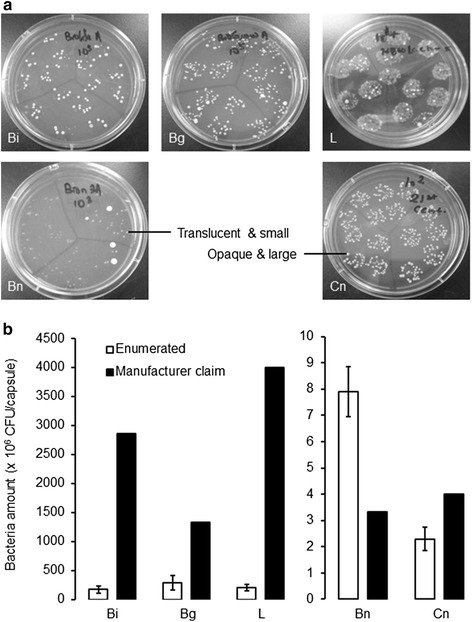
The recovery and enumeration of probiotic bacteria from dietary supplements. (**a**) Representative MRS agar plates of probiotics bacteria recovered from Bi, Bg, L, Bn and Cn dietary supplements showing different colony morphologies and densities. (**b**) Comparison of the enumerated probiotics bacteria to that claimed by the manufacturers of the respective products (Table 1). Error bars represent standard error of the mean (*n* = 2)

Viable bacteria were recovered on MRS media and their amounts were enumerated prior to antibiotic susceptibility tests. Probiotic bacteria, particularly the *Lactobacilli* strains, from all brands of dietary supplements were successfully recovered on MRS agar (Fig. 1a). The recovered bacteria have different colony morphologies of which two are particularly distinctive. One, a larger sized opaque colony is present in all brands at similar amounts to the other colony type that is smaller in size and translucent, but appears to be far fewer in brands Bn and L respectively (Fig. 1a). This suggests that there is more than one type of probiotic strain in the respective products and this is consistent with the claims of the manufacturers (Table 1). The bacteria enumeration revealed that with the exception of brand Bn, samples from all brands have bacteria amounts that are fewer than that claimed by their manufacturers (Fig. 1b). Most significant of these are samples from brands Bi, Bg and L which have viable bacteria of only 6 %, 22 % and 3 % of the amounts stated on their respective datasheets. The bacteria concentration of brand Cn is 57 % of that claimed by the manufacturer while brand Bn has a surprising bacteria count that is more than double the stated amount. The enumerated bacteria amounts were however above the recommended minimum threshold of 10^6^ CFU/capsule [17]. The overestimation of probiotic amounts in four of the five products tested suggest that 1) a significant amounts of bacteria have been compromised during the processing stages which may include improper handling and storage conditions or 2) the manufacturers have deliberately overestimated the probiotic content of the products in an attempt to compete for consumers [18]. This finding seems to agree with other reports concerning mislabeling, overestimation and misidentification of probiotics in various food and health products [19]. It is therefore doubtful that the health claims of the respective products can be achieved by the inferior amounts of viable probiotic bacteria although some reports have claimed that viability is not essential and that probiotic DNA or cell wall materials may be sufficient to confer certain health effects [20]. This enumeration provides useful preliminary data shedding light on the accuracy and reliability of the probiotic bacteria information detailed on the datasheet of dietary supplements as regulations and legislations concerning product labeling are currently lacking [21].

**Fig. 2:**
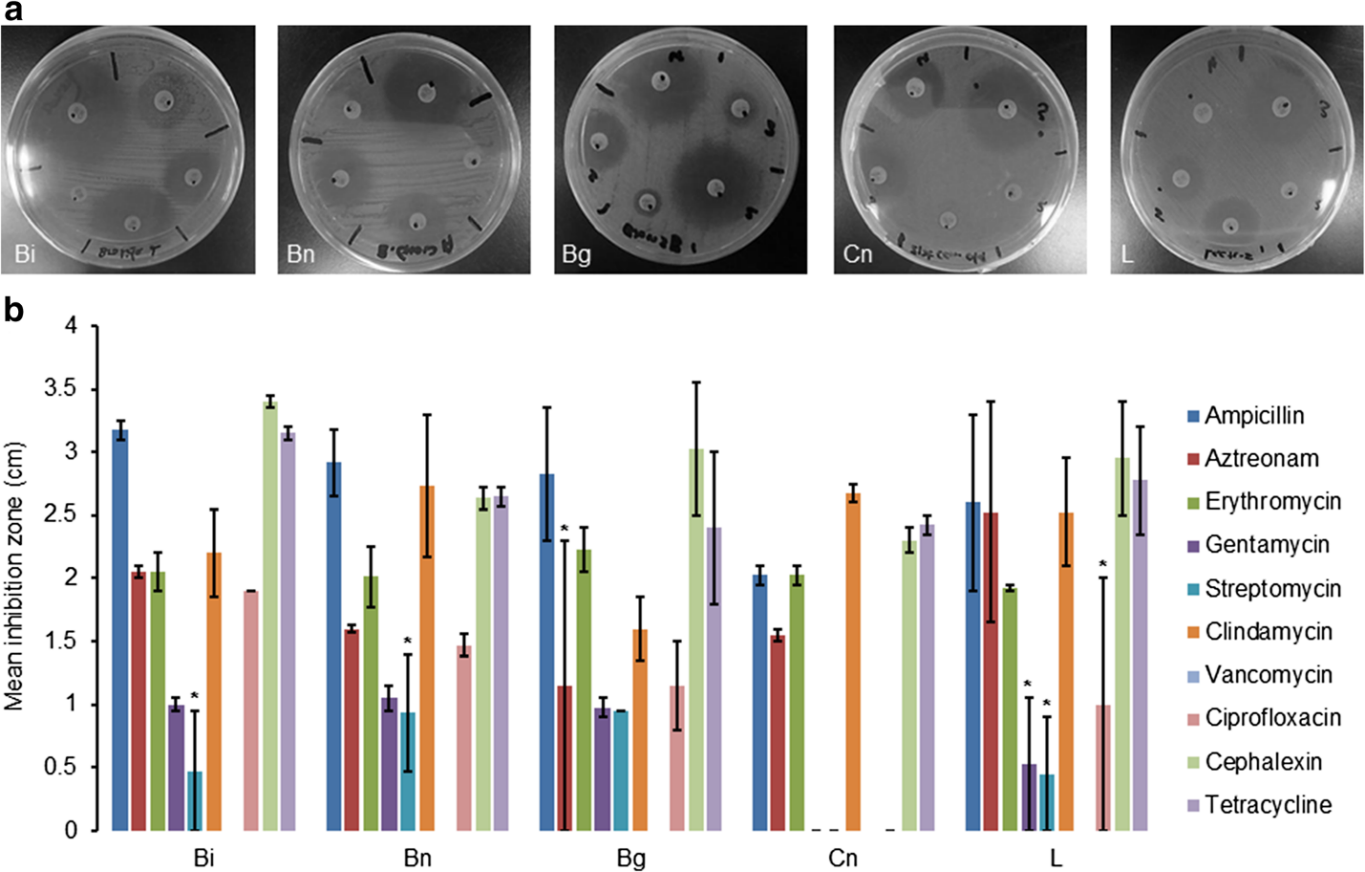
The antibiotic susceptibility profile of probiotic bacteria in the dietary supplements. (**a**) Representative MRS agar plates of bacteria lawn of Bi, Bn, Bg, Cn and L dietary supplements layered with antibiotic discs showing susceptibility towards multiple antibiotics as characterized by the presence of ‘clear’ inhibition zones. (**b**) Mean inhibition zones measured from the bacteria lawn of Bi, Bn, Bg, Cn and L dietary supplements layered with the respective antibiotic discs. Error bars represent standard error of the mean (*n* ≥ 2) and (*) represents inhibition zone present in only certain batches of bacteria in the respective dietary supplement

In the antibiotic resistant screening, clear inhibition zones of > 0.5 cm were measured from the bacteria lawn of isolates from all batches of brands Bi, Bn, Bg, Cn and L towards ampicillin, erythromycin, clindamycin, cephalexin and tetracycline antibiotics (Fig. 2b), suggesting no resistance towards these antibiotics. Meanwhile, inhibition zones detected in only certain batches of the probiotic products were that of brands Bi and Bn, Bg and L in the presence of the respective streptomycin, aztreonam and gentamycin, streptomycin and ciprofloxacin antibiotics (Fig. 2b). This batch-to-batch variation implies that these resistances were not conferred by intrinsic genes but more likely a result of acquired mobile genetic elements by a transfer event and this is a concern because mobile elements such as plasmids and transposons can be transferred from one cell to another by conjugation [10]. Indeed, this mechanism has been demonstrated *in vitro* where antibiotic resistant gene transferred from one *Lactobacillus* to another and more worryingly, also from *Lactobacilli* to other species including pathogenic strains such as *Staphylococcus* [13] and vice versa [22]. Streptomycin and gentamycin belong to the aminoglycosides and resistance towards this group of antibiotic may be conferred by intrinsic *Lactobacilli* aminoglycoside resistant genes *aac(6’)-aph(2”), ant(6),* and *aph(3’)-IIIa* respectively [11]. Consistent with this speculation, bacteria of brand Cn also showed no inhibition zone against the gentamycin and streptomycin antibiotics besides also being resistant towards ciprofloxacin (Fig. 2b). While resistance towards aztreonam, a derivative of the beta-Iactam antibiotics, may be conferred by the *blaZ* genes which have also been previously found in *Lactobacilli* [2], detection of ciprofloxacin resistance could not be attributed to known fluoroquinolone resistance genes as they have not been identified in *Lactobacilli*. Although probiotic bacteria (predominantly *Lactobacilli*) of all products were able to grow in the presence of vancomycin (Fig. 2b), this is attributed to intrinsic vancomycin resistant genes in *Lactobacilli* strains rather than a transfer event. This intrinsic resistant gene prevents vancomycin binding at the cytoplasmic end of their cell walls due to replacement of the terminal amino acid residue [23]. Notably, tetracycline and erythromycin resistance were not detected in isolates of all brands although genes conferring resistance towards these classes of antibiotics (i.e. the tetracycline and macrolides) are well-characterized in *Lactobacilli* [24]. Although reports on antibiotic resistance in probiotic isolates of dietary supplements are elusive, such data from foods [12] and biological sources [25] are widely reported [6, 9, 16]. Further, resistant determinants have also been identified with the resistance genes of tetracycline (>10), aminoglycoside (at least 4) and beta-lactam [12] constituting an expanding list of genetic elements in *Lactobacilli*.

In summary, this is a pilot study that reports the present of antibiotic resistance in probiotic bacteria in dietary supplements thus expanding the screening of antibiotic resistance to include this largely unexplored but increasingly popular group of functional foods. This study also serves as a platform for further screenings and in-depth characterization of the resistant determinants and the molecular machinery that confers the resistance. In particular, resistant probiotics can be isolated and identified by a combination of biochemical tests and 16S rRNA gene sequencing analysis and the antibiotic resistant genes characterized using degenerate primers and/or a motif-based identification approach [26]. Manufacturing companies of dietary supplements should incorporate antibiotic susceptibility screening using diagnostic tools such as microarray chips and PCR approaches in the production chain. Additionally, curative strategies such as the removal of genetic elements that harbor antibiotic resistance could also be applied to the relevant probiotic strains. The latter has been applied to the probiotic *L. reuteri* DSM 17938 whereby two resistant plasmids were successfully removed from the parent *L. reuteri* (ATCC 55730) [27] while not affecting the probiotic properties of the strain.

## Additional file

Additional file 1:
**Extended Materials and Methods.**

## References

[1] Kaur IP, Chopra K, Saini A (2002). Probiotics: potential pharmaceutical applications. Eur J Pharm Sci.

[2] Perdigon G, Fuller R, Raya R (2001). Lactic acid bacteria and their effect on the immune system. Curr Issues Intest Microbiol.

[3] Hirayama K, Rafter J (2000). The role of probiotic bacteria in cancer prevention. Microbes Infect.

[4] Kechagia M, Basoulis D, Konstantopoulou S, Dimitriadi D, Gyftopoulou K, Skarmoutsou N (2013). Health benefits of probiotics: a review. ISRN Nutr.

[5] Stanton C, Gardiner G, Meehan H, Collins K, Fitzgerald G, Lynch PB (2001). Market potential for probiotics. Am J Clin Nutr.

[6] Sharma P, Tomar SK, Goswami P, Sangwan SR (2014). Antibiotic resistance among commercially available probiotics. Food Res Int.

[7] Markets and Markets: In: Probiotic market-advanced technologies and global market (2009–2014). 2009. http://www.marketsandmarkets.com/Market-Reports/probiotic-market-advanced-technologies-and-global-market-69.html. Accessed 28 January 2015.

[8] Homayouni A, Alizadeh M, Alikhah H, Zijah V: Functional Dairy Probiotic Food Development: Trends, Concepts, and Products. In: Everlon Rigobelo, editor. Probiotics. InTech 2012. doi:10.5772/48797. 2012

[9] Mathur S, Singh R (2005). Antibiotic resistance in food lactic acid bacteria-a review. Int J Food Microbiol.

[10] Broaders E, Gahan CG, Marchesi JR (2013). Mobile genetic elements of the human gastrointestinal tract: potential for spread of antibiotic resistance genes. Gut Microbes.

[11] Devirgiliis C, Zinno P, Perozzi G (2013). Update on antibiotic resistance in foodborne *Lactobacillus* and *Lactococcus* species. Front Microbiol.

[12] Gueimonde M, Sanchez B, G de Los Reyes-Gavilan C, Margolles A (2013). Antibiotic resistance in probiotic bacteria. Front Microbiol.

[13] Tannock GW, Luchansky JB, Miller L, Connell H, Thode-Andersen S, Mercer AA (1994). Molecular characterization of a plasmid-borne (pGT633) erythromycin resistance determinant (*ermGT*) from *Lactobacillus reuteri* 100*–*63. Plasmid.

[14] Mater DDG, Langella P, Corthier G, Flores MJ (2008). A Probiotic *Lactobacillus* strain can acquire vancomycin resistance during digestive transit in mice. J Mol Microbiol Biotechnol.

[15] Munsch-Alatossava P, Alatossava T (2007). Antibiotic resistance of raw-milk-associated psychrotrophic bacteria. Microbiol Res.

[16] Sharma P, Tomar SK, Sangwan V, Goswami P, Singh R: Antibiotic resistance of *Lactobacillus sp.* isolated from commercial probiotic preparations. J Food Safety 2015, doi:10.1111/jfs.12211.

[17] Shah NP (2000). Probiotic bacteria: selective enumeration and survival in dairy foods. J Dairy Sc.

[18] Farnworth ER (2008). The evidence to support health claims for probiotics. J Nutr.

[19] Hamilton-Miller JMT, Shah S, Winkler JT (1999). Public health issues arising from microbiological and labelling quality of foods and supplements containing probiotic microorganisms. Public Health Nutr.

[20] Lahtinen SJ: Probiotic viability - does it matter? Microb Ecol Health D 2012, 23:doi:10.3402/mehd.v23i0.18567. 10.3402/mehd.v23i0.18567 PMC3747757 23990833

[21] Przyrembel H (2001). Consideration of possible legislation within existing regulatory frameworks. Am J Clin Nutr.

[22] Vescovo M, Morelli L, Bottazzi V, Gasson MJ (1983). Conjugal transfer of broad-host-range plasmid pAMbeta1 into *Enteric* species of lactic acid bacteria. Appl Environ Microbiol.

[23] Delcour J, Ferain T, Deghorain M, Palumbo E, Hols P (1999). The biosynthesis and functionality of the cell-wall of lactic acid bacteria. Antonie Van Leeuwenhoek.

[24] Zonenschain D, Rebecchi A, Morelli L (2009). Erythromycin- and tetracycline-resistant *Lactobacilli* in Italian fermented dry sausages. J Appl Microbiol.

[25] Ocana V, Silva C, Nader-Macias ME (2006). Antibiotic susceptibility of potentially probiotic vaginal *Lactobacilli*. Infect Dis Obstet Gynecol.

[26] Wong A, Gehring C (2013). Computational identification of candidate nucleotide cyclases in higher plants. Methods Mol Biol.

[27] Rosander A, Connolly E, Roos S (2008). Removal of antibiotic resistance gene-carrying plasmids from *Lactobacillus reuteri* ATCC 55730 and characterization of the resulting daughter strain, *L. reuteri* DSM 17938. Appl Environ Microbiol.

