# Supplementary material for: Detection of antibiotic resistance in probiotics of dietary supplements
Source: Nutr J. 2015 Sep 14;14:95. doi: 10.1186/s12937-015-0084-2 (PMC4568587; doi:10.1186/s12937-015-0084-2)
Supplement: Additional file 1: — Extended Materials and Methods. [file 12937_2015_84_MOESM1_ESM.docx]

**Additional file 1**

**Extended Materials and Methods**

*Probiotic dietary supplements, antibiotic discs and microorganisms*

Five brands of probiotic dietary supplements designated here as Bi, Bn, Bg, Cn and L, were purchased from local pharmacies or retail outlets, and their details are as listed in Table 1. These products are commonly available and contain relatively high amounts of probiotic bacteria. The ampicillin, aztreonam, erythromycin, gentamycin, streptomycin, clindamycin, vancomycin, ciprofloxacin, cephalexin and tetracycline antibiotic discs were purchased from HiMedia, India, and they represent different classes and have contrasting mode of actions (Table 2).

*Recovery and enumeration of probiotic bacteria*

One randomly chosen capsule of the respective probiotic dietary supplements was dissolved in sterile double distilled water [16] and diluted accordingly. The de Man, Rogosa and Sharpe (MRS) media (Difco, USA) was used in this study and since this media is selective for *Lactobacilli*, other probiotic strains such as *Bifidobacteria* and *Streptococcus* were excluded from the bacteria count and not considered in subsequent antibiotic resistance screening. One capsule contains equal amounts of each probiotic strain. In products where such information is not stated, equal contributions from each probiotic content is assumed. The concentration of probiotic bacteria in the supplements were enumerated using drop plate method as described by Munsch-Alatossava & Alatossava (2007) [15]. Briefly, the MRS agar plate was divided into three equal-sized quadrants and 5 drops of 10 µL each of the appropriately diluted samples was dropped onto one quadrant. The same was repeated for the remaining two quadrants so that bacteria enumeration of each sample was performed in triplicates. The plates were incubated under aerobic conditions at 37 ºC for 48 hours after which, the number of colonies was enumerated and colony morphology observed. Only colony counts between 25 and 300 were considered to be representative of the sample and therefore, used for bacteria concentration calculations which were expressed in colony-forming units (CFU) per capsule of the respective dietary supplement. The enumerated viable bacteria concentrations were compared to that claimed by the manufacturers (Table 1).

*Screening for antibiotic resistant probiotics*

The dissolved samples (10^6^ CFU) were cultured overnight in MRS broth for enrichment of probiotic bacteria after which the overnight culture was adjusted to uniform concentrations of 7x10^6^ CFU/mL of bacteria by spectroscopy (OD_690nm_) prior to antibiotic susceptibility tests using commercial antibiotic discs and according to the manufacturer’s instructions. A total of 100 µL of standardized probiotic suspension (7x10^6^ CFU/mL) was streaked onto MRS agar using a sterile cotton swab until homogeneity. Then, antibiotic discs (HiMedia, India) (Table 2) were layered onto the bacteria lawn in each quadrant of the agar and the plates incubated under aerobic conditions at 37 ºC for 24 hours. Any inhibition (clear) zones surrounding the antibiotic discs were noted and their diameters were measured. The antibiotic susceptibility test was repeated on a different batch of the same dietary supplements and the results from all the batches were averaged. Probiotics were considered susceptible if the inhibition zone diameter is > 0.5 cm. To determine the effectiveness of the antibiotics discs, two reference microorganisms *Escherichia coli* and *Staphylococcus aureus* (Microbiology Laboratory of UCSI University, Malaysia) were tested for susceptibility to the respective antibiotics (Table S1).

**Table 1** Antibiotic susceptibility profile of reference bacteria

| Antibiotic | Inhibition zone (cm ± SD) | |
| --- | --- | --- |
|  | *E. coli* ATCC 25922 | *S. aureus* ATCC 25923 |
| Ampicillin (10 mcg) | 0.00 ± 0.00 | 3.45 ± 0.07 |
| Aztreonam (30 mcg) | 3.15 ± 0.07 | 1.45 ± 0.07 |
| Erythromycin (15 mcg) | 1.05 ± 0.07 | 2.00 ± 0.00 |
| Gentamicin (5 mcg) | 1.45 ± 0.07 | 1.80 ± 0.00 |
| Streptomycin (10 mcg) | 1.95 ± 0.21 | 2.05 ± 0.07 |
| Clindamycin (2 mcg) | 1.65 ± 0.07 | 2.15 ± 0.07 |
| Vancomycin (30 mcg) | 1.15 ± 0.07 | 1.70 ± 0.00 |
| Cephalexin (30 mcg) | 0.00 ± 0.00 | 3.70 ± 0.00 |
| Tetracycline (30 mcg) | 1.45 ± 0.07 | 3.55 ± 0.07 |
| Ciprofloxacin (10 mcg) | 3.70 ± 0.00 | 3.00 ± 0.00 |
